# Investigation of Machining Characteristics in Electrical Discharge Machining Using a Slotted Electrode with Internal Flushing

**DOI:** 10.3390/mi14111989

**Published:** 2023-10-27

**Authors:** Minghao Gao, Ming Liu, Jianqing Han, Qinhe Zhang

**Affiliations:** 1 Key Laboratory of High Efficiency and Clean Mechanical Manufacture (Ministry of Education), School of Mechanical Engineering, Shandong University, Jinan 250061, China; 2 National Demonstration Center for Experimental Mechanical Engineering Education, Shandong University, Jinan 250061, China

**Keywords:** electrical discharge machining, slotted electrode, internal flushing, machining characteristics

## Abstract

In die-sinking electrical discharge machining (EDM), it is challenging to implement internal flushing, mainly because it is easy to produce residual material columns on the workpiece cavity’s bottom surface, affecting the processing quality and efficiency. In order to solve this problem, the internal flushing slotted electrode EDM technology was proposed. The slotted electrode was designed, and its preparation method was described. The influence of pulse width, pulse interval, and flushing pressure on the performance of the internal flushing slotted electrode EDM was studied using single-factor experiments. The experimental results indicate that, with the increase in pulse width, the material removal rate (MRR) increases first and then decreases, while the electrode wear rate (EWR) and the relative electrode wear rate (REWR) decrease gradually; with the increase in pulse interval, the MRR decreases, while the EWR and the REWR increase gradually; with the increase in flushing pressure, the MRR increases first and then decreases, while the EWR and the REWR increase gradually. When the slotted electrode is used for continuous internal flushing EDM, the appropriate pulse width, flushing pressure, and smaller pulse interval can improve the MRR and reduce the EWR and the REWR.

## 1. Introduction

EDM is a non-contact, non-traditional machining technology that is widely used in die and aerospace industries. Because it does not use any macroscopic cutting force in the machining process and is not sensitive to the mechanical properties of materials, EDM has unique advantages in processing conductive and difficult-to-cut materials [1,2,3,4]. Many factors affect the performance of EDM, such as the chemical and physical properties of the tool electrode and the workpiece material [5,6,7,8], the electrical processing parameters [9,10], and the type of processing media [11]. The most critical factor is whether the erosion products can be discharged from the discharge gap in time. Suppose the erosion products in the discharge gap cannot be discharged in time. In that case, arc discharge and short circuits will frequently occur during the machining process, affecting the gap discharge state and processing efficiency and restricting the development of EDM in the direction of high efficiency [12,13].

In order to solve this problem, scholars have conducted many studies and proposed many new beneficial measures, such as applying flushing fluid [14,15], ultrasonic-vibration-assisted machining [16,17,18], electrode jump motion [19,20], electrode rotation motion [21], and external-magnetic-field-assisted machining [22]. In recent years, some scholars have proposed the slotted electrode, which can effectively discharge the erosion products, and studied the effect of the slotted electrode on the performance of EDM. Puthumana et al. [23] studied the efficiency of the slotted electrode in dry EDM. They used the Taguchi Method to explore the optimal conditions for processing parameters. They found that the four-slotted electrode can provide optimal EDM efficiency and that using slotted electrodes significantly reduces the electrode wear rate. Jie-Shing Lo et al. [24] investigated the use of slotted electrodes to improve the removal of erosion products. They analyzed the waveforms of machining voltage and current signals to gain the electrical discharge condition of EDM. Compared with general cylindrical, non-slotted electrodes, the deep-slotted electrodes improved the MRR on large-scale and hemisphere EDM results by 91% and 116.7%, respectively.

For non-rotating electrode die-sinking EDM, flushing is one of the most effective means to remove erosion products. Compared to external flushing, internal flushing is more effective. In traditional EDM with internal flushing, it is necessary to design a flow channel for the working fluid to enter and exit the electrode. Therefore, sizeable residual material columns are often formed on the bottom surface of the workpiece, which need to be removed through secondary processing, reducing the processing efficiency. In order to improve the internal flushing EDM, Li et al. [25] studied the bunched electrode EDM. The solid electrodes with complex end shapes were dispersed into many tubular electrodes bunched together to obtain an electrode with an approximate end shape. The structure inside the bunched electrode can achieve a uniform internal flow field, which is especially suitable for high-energy rough machining. Jiang et al. [26] studied the porous electrode EDM. A large number of copper particles were sintered at high temperatures to prepare an electrode with a porous structure. The flow channel formed by disordered pores inside the porous electrode can realize three-dimensional and distributed internal flushing, which is conducive to discharge erosion products. A tiny pulse interval can be applied and a high MRR can be achieved due to the high-duty cycle. However, the forming accuracy of the bunched and porous electrodes is poor, making the allowance of subsequent processing extensive. In the finishing stage, it is necessary to prepare solid electrodes again for trimming processing, which is more complicated.

In this study, the technology of combining internal flushing and slotted electrodes was proposed as an internal flushing slotted electrode EDM. It integrates the advantages of the internal flushing EDM and the slotted electrode EDM to improve machining performance and avoid the sizeable residual material columns during processing. The fresh working fluid is transported to the discharge gap through the narrow slit inside the slotted electrode to promote the fluidity of the working fluid in the discharge gap, thereby promoting the discharge of the electrical erosion products in the discharge gap, improving the processing stability and efficiency. Due to the narrow slit of the slotted electrode, the resulting material columns can be easily removed using the translational motion function of the machine tool. In this study, in order to explore the influence of various processing parameters on the machining performance in the internal flushing slotted electrode EDM, the internal flushing slotted electrode was designed, and its preparation method was elaborated. The single-factor experiments of slotted electrodes were carried out to study the effects of pulse width, pulse interval, and flushing pressure on MRR, EWR, and REWR. Applying the slotted electrode with internal flushing in EDM can reduce EWR and REWR, and selecting appropriate flushing pressure can improve MRR, which provides a new idea for solving the problem of the residual material columns generated during machining. This study contributes to expanding the application of internal flushing in non-rotating electrodes, promoting the development of EDM in the manufacturing industry to a certain extent. In particular, it provides a reference for solving the problem that the flushing effect in the central flushing hole is not ideal when the electrode shape is irregular and the area is large.

## 2. Materials and Methods

### 2.1. Preparation Method of Slotted Electrode

**Figure 1:**
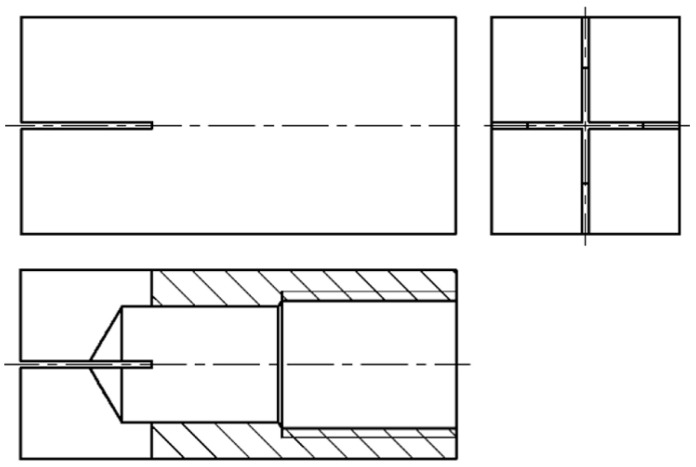
The structure of the slotted electrode.

The rectangular block electrodes of 13 mm × 15 mm × 30 mm were used to prepare the slotted electrodes, and the electrode material used was red copper. The structure of the slotted electrode is shown in Figure 1. A number of narrow slits with a certain depth are cut from the end of the solid electrode to the inside of the electrode, and the central liquid flushing hole is processed at the opposite end of the electrode so that the narrow slit is connected with the liquid flushing hole and the working fluid can flow inside it. The depth of the central flushing hole is 23 mm, and the diameter is 8 mm. The two narrow slits are perpendicular to each other and perpendicular to the side of the electrode, respectively, and the width of the narrow slits is 0.5 mm. In order to make the inlet and outlet area of the narrow slits approximately equal, the depth of the narrow slits is 9 mm.

**Figure 2:**
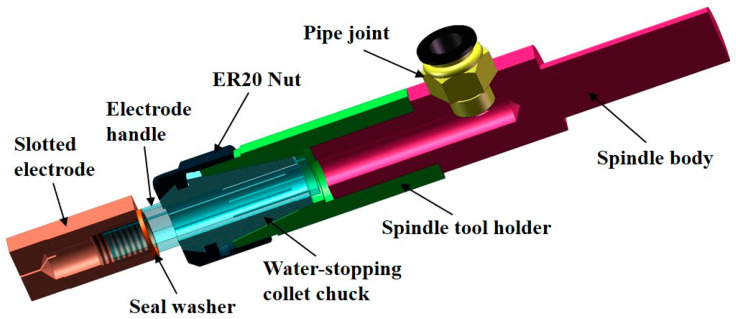
The schematic diagram of the internal flushing main spindle.

The internal flushing main spindle is connected to the spindle of the EDM machine tool through the spindle body. The schematic diagram of the internal flushing main spindle is shown in Figure 2. In order to facilitate the loading and unloading of the electrode, a thread is processed at the top of the flushing hole in the center of the electrode.

**Figure 3:**
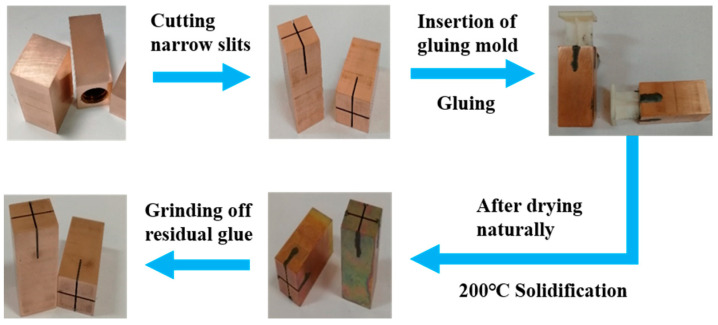
The preparation process of the slotted electrode.

The preparation process of the slotted electrode is shown in Figure 3. In order to prevent the swarf from blocking the slit when processing the central flushing hole, the cutting of the slit must be placed in the last process during preparation. The discharge gap at the bottom of the electrode is the central processing area of EDM. In order to improve the discharge effect of the erosion products, the working fluid must flow into the discharge gap. Therefore, it is necessary to seal the lateral slits on the side of the electrode. In this study, the graphite-based heat-resistant conductive adhesive (NGWD-1400K, Zibo Nengxu High Temperature Products Sales Co., Ltd., Zibo, China) was used to seal the lateral slit of the electrode. The adhesive can ensure its synchronous discharge wear with the copper-based electrode. The corresponding gluing mold was designed and printed to ensure the uniform thickness of the adhesive layer (1 mm) and the consistency of the sealed electrode.

### 2.2. Experimental Platform

**Figure 4:**
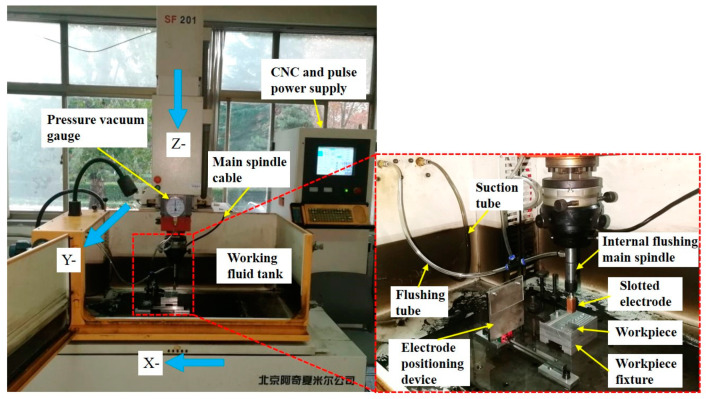
Slotted electrode EDM experiment platform.

The experiments were carried out using an EDM machine tool (Beijing Agie Charmilles, Beijing, China). The internal flushing spindle was clamped on the machine’s main spindle and connected to the flushing tube through a quick-connector to realize the internal flushing EDM. The specific experimental platform is shown in Figure 4.

Because the workpiece needs to translate the square trajectory of the machine tool during the machining process, in order to ensure that the standard rectangular hole is processed on the workpiece, a special electrode positioning device and a workpiece fixture must be designed to align the electrode and the workpiece with the machine bed. In the electrode positioning device, an insulating organic glass plate needs to be attached to the surface of the electrode positioning plate to prevent the contact between the electrode and the electrode positioning plate from triggering the contact perception of the machine tool.

### 2.3. Experiment Setup

In the experiments, the working fluid is kerosene-based EDM oil, and the workpiece material is high-speed steel (W9Mo3Cr4V). The chemical composition of high-speed steel is shown in Table 1. For convenience of clamping, the workpiece is cut into a small cuboid of 25 mm× 22 mm× 10 mm. In the machining process, the workpiece translates with the square trajectory of the machine tool, and the translational radius is 0.5 mm; the electrode moves periodically with the main spindle of the machine tool in a lifting motion, with a height of 1 mm.

In order to explore the influence of various processing parameters on the machining performance in the internal flushing slotted electrode EDM, the single-factor experiments were designed by selecting the pulse width, pulse interval, and flushing pressure as variables. In order to determine the appropriate parameter range, the MRR, EWR, and REWR were observed during pre-experiments. Because the optional machining parameter values of the EDM machine tool are discrete, the processing effect is better when the peak current is set to 25.6 A and the open circuit voltage and reference voltage are set to 100 V and 52 V. Therefore, the values of each factor level of the single-factor experiments were determined, as shown in Table 2. In the process of EDM, the electrode feeds down 4 mm along the *Z* axis from the surface of the workpiece.

The MRR, EWR, and REWR were selected as the evaluation indexes of processing performance. The calculation formulae are as follows:

(1) $$\mathrm{MRR}=\frac{M_{wi}-M_{wj}}{\rho_{w}\cdot t}\left({\mathrm{mm}}^{3}/\min\right)$$

(2) $$\mathrm{EWR}=\frac{M_{ei}-M_{ej}}{\rho_{e}\cdot t}\left({\mathrm{mm}}^{3}/\min\right)$$

(3) $$\mathrm{REWR}=\frac{\mathrm{EWR}}{\mathrm{MRR}}\times 100\%$$

where *M_wi_* and *M_wj_* denote the masses (g) of the workpiece before and after processing, respectively; *M_ei_* and *M_ej_* denote the masses (g) of the electrode before and after processing, respectively, obtained by a high precision electronic balance (Shimadzu AUW220D, Kyoto City, Japan) with an accuracy of 0.1 mg; *ρ_w_* denotes the workpiece density (g/mm^3^), taken as 7.835 × 10^−3^; *ρ_e_* denotes the electrode density (g/mm^3^), taken as 8.9 × 10^−3^; and *t* denotes the processing time (min) obtained using the machine’s processing timing function.

## 3. Results and Discussion

### 3.1. Effect of Pulse Width on the Processing Performance

**Figure 5:**
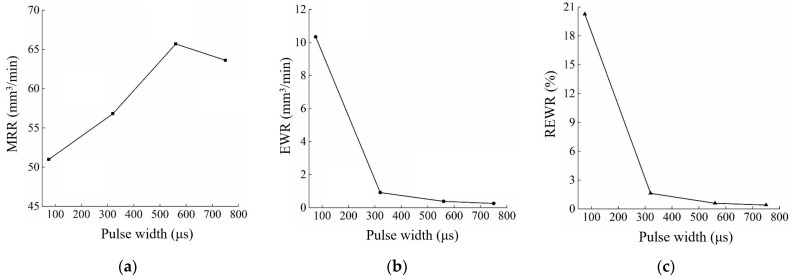
Effect of pulse width on (**a**) MRR, (**b**) EWR, and (**c**) REWR. (Pulse width: 75 μs, 320 μs, 560 μs, 750 μs; pulse interval: 24 μs; flushing pressure: 5 kPa; peak current: 25.6 A; open circuit voltage: 100 V; reference voltage: 52 V).

**Figure 6:**
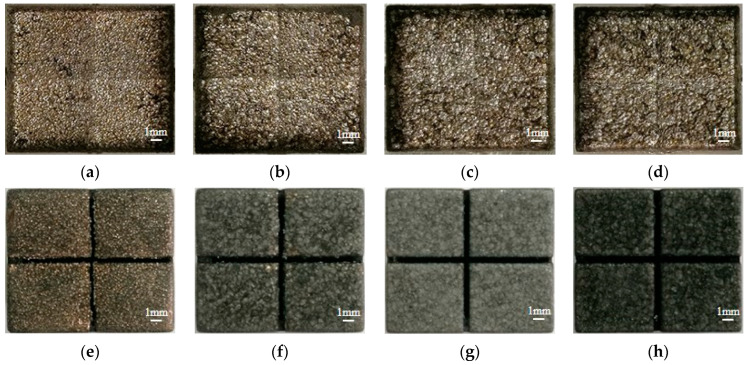
The surface of the workpiece cavity after processing under different pulse widths. (**a**) 75 μs; (**b**) 320 μs; (**c**) 560 μs; (**d**) 750 μs. And the surface of the electrode after processing under different pulse widths. (**e**) 75 μs; (**f**) 320 μs; (**g**) 560 μs; (**h**) 750 μs.

The influence of pulse width on MRR, EWR, and REWR is shown in Figure 5. With the increase in pulse width, the MRR increases first and then decreases, and the EWR and the REWR decrease. The surface of the workpiece cavity and electrode after processing under different pulse widths is shown in Figure 6.

The increase in the pulse width leads to the increase in the discharge energy of a single pulse, the volume of the workpiece removed per unit of time increases, and the generated pits become larger and deeper. So, the MRR increases. However, as the pulse width increases, the discharge channel also expands. The energy dispersion is more prominent, and the volume of the material removed is almost unchanged. Since the discharge time increases, the MRR decreases.

**Figure 7:**
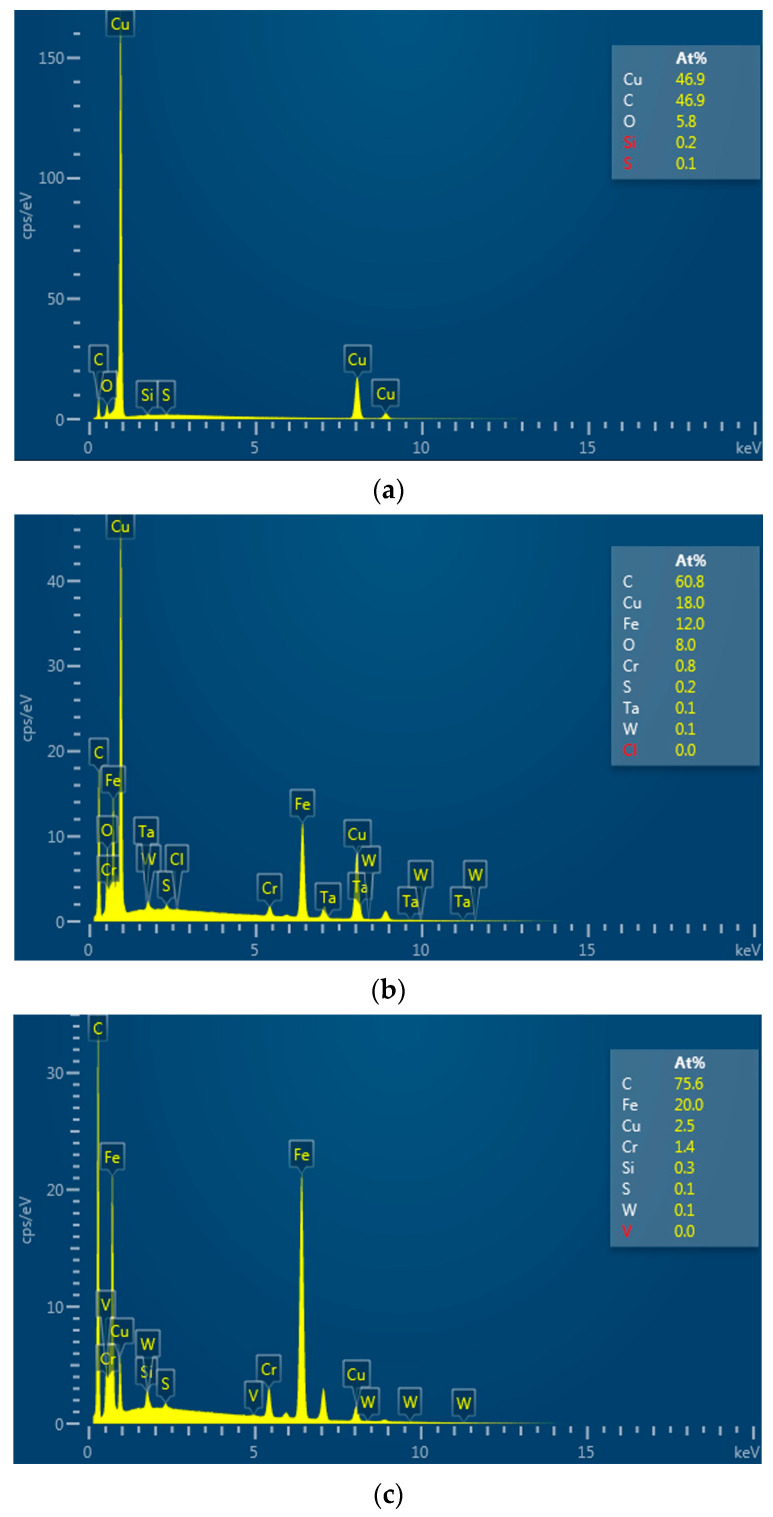
EDS (Oxford Instruments X-MAX, Abingdon, UK) analysis of the electrode surface (**a**) before processing and the electrode surface processed under a pulse width of (**b**) 75 μs and (**c**) 320 μs.

It can be seen in Figure 7 that, when the pulse width is 75 μs, the atomic percentage of carbon on the surface of the processed electrode increases by 13.9% compared with that before processing. When the pulse width increases from 75 μs to 320 μs, the atomic percentage of carbon on the surface of the processed electrode increases by 14.8%. Increasing the pulse width means increasing the discharge time per unit of time, resulting in an increase in the temperature of the working liquid between the electrodes and the concentration of carbon particles. This helps the carbon particles to adsorb on the electrode surface (positive electrode) to form a high-temperature carbon black film, thereby reducing the EWR and the REWR. From Figure 6e, when the pulse width is 75 μs, the carbon black film on the bottom of the electrode is incompletely formed, exposing a large amount of copper matrix. When the pulse width increases to 320 μs, the bottom of the electrode is completely covered by the carbon black film, which increases the wear resistance of the electrode. In addition, it can be seen from Figure 6a that, when a small pulse width is used for processing, due to the significant electrode wear, the workpiece cavity’s bottom surface becomes uneven, seriously affecting the processing quality. Therefore, when the slotted electrode is used for continuous internal flushing processing, the appropriate pulse width should be adopted to ensure processing efficiency and reduce electrode wear.

### 3.2. Effect of Pulse Interval on the Processing Performance

**Figure 8:**
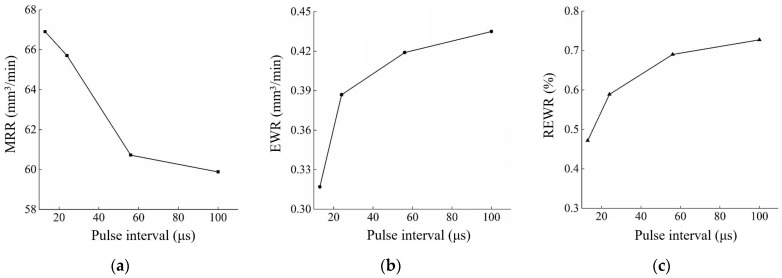
Effect of pulse interval on (**a**) MRR, (**b**) EWR, and (**c**) REWR. (Pulse interval: 13 μs, 24 μs, 56 μs, 100 μs; pulse width: 560 μs; flushing pressure: 5 kPa; peak current: 25.6 A; open circuit voltage: 100 V; reference voltage: 52 V).

**Figure 9:**
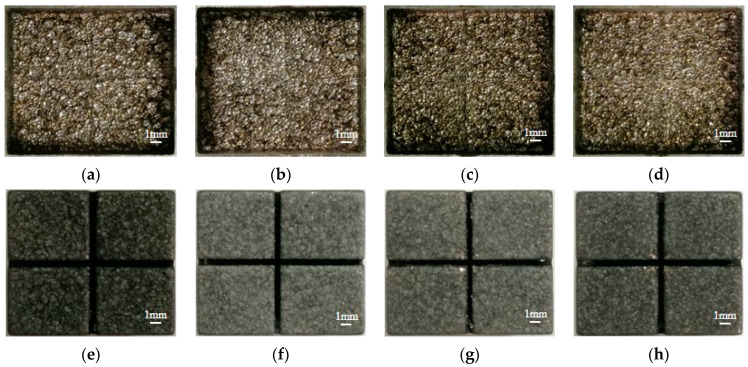
The surface of the workpiece cavity after processing under different pulse intervals. (**a**) 13 μs; (**b**) 24 μs; (**c**) 56 μs; (**d**) 100 μs. And the surface of the electrode after processing under different pulse intervals. (**e**) 13 μs; (**f**) 24 μs; (**g**) 56 μs; (**h**) 100 μs.

The effect of the pulse interval on MRR, EWR, and REWR is shown in Figure 8. With the increase in the pulse interval, the MRR gradually decreases, and the EWR and the REWR gradually increase. The surface of the workpiece cavity and electrode after processing under different pulse intervals is shown in Figure 9.

The increase in pulse interval causes a lot of wasted processing time, resulting in decreased MRR. Meanwhile, the increase in pulse interval decreases the discharge time per unit of time. Thus, reducing the number of carbon particles affects the formation of the carbon black film. It reduces the wear resistance of the electrode and increases the EWR and the REWR. It can be seen from Figure 9e–h that the carbon black film on the bottom of the electrode becomes incomplete with the increase in pulse interval. At a pulse interval of 56 μs, the copper matrix is already exposed on the middle of the electrode bottom surface. Therefore, a smaller pulse interval should improve the processing efficiency and reduce electrode wear when the slotted electrode is used for continuous internal flushing processing.

### 3.3. Effect of Flushing Pressure on the Processing Performance

**Figure 10:**
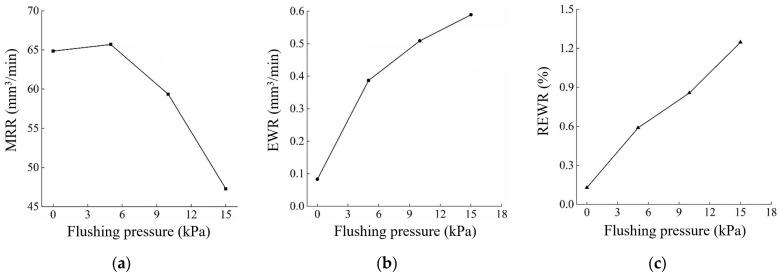
Effect of flushing pressure on (**a**) MRR, (**b**) EWR, and (**c**) REWR. (flushing pressure: 0, 5 kPa, 10 kPa, 15 kPa; Pulse width: 560 μs; pulse interval: 24 μs; peak current: 25.6 A; open circuit voltage: 100 V; reference voltage: 52 V).

**Figure 11:**
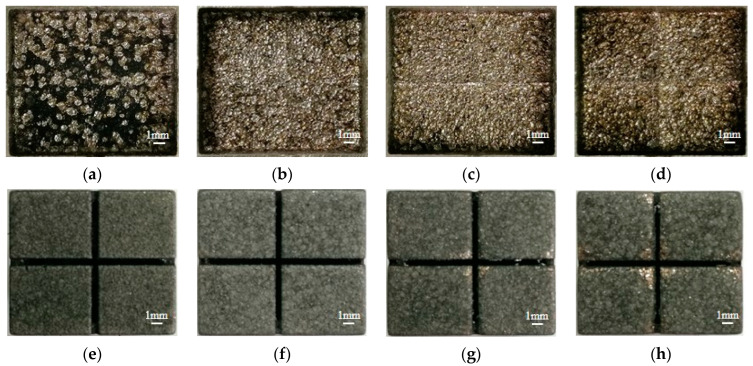
The surface of the workpiece cavity after processing under different flushing pressures. (**a**) 0; (**b**) 5 kPa; (**c**) 10 kPa; (**d**) 15 kPa. And the surface of the electrode after processing under different flushing pressures. (**e**) 0; (**f**) 5 kPa; (**g**) 10 kPa; (**h**) 15 kPa.

The effect of flushing pressure on the MRR, EWR, and REWR is shown in Figure 10. With the increase in flushing pressure, the MRR increases first and then decreases, while the EWR and REWR increase gradually. The surface of the workpiece cavity and electrode after processing under different flushing pressures is shown in Figure 11.

**Figure 12:**
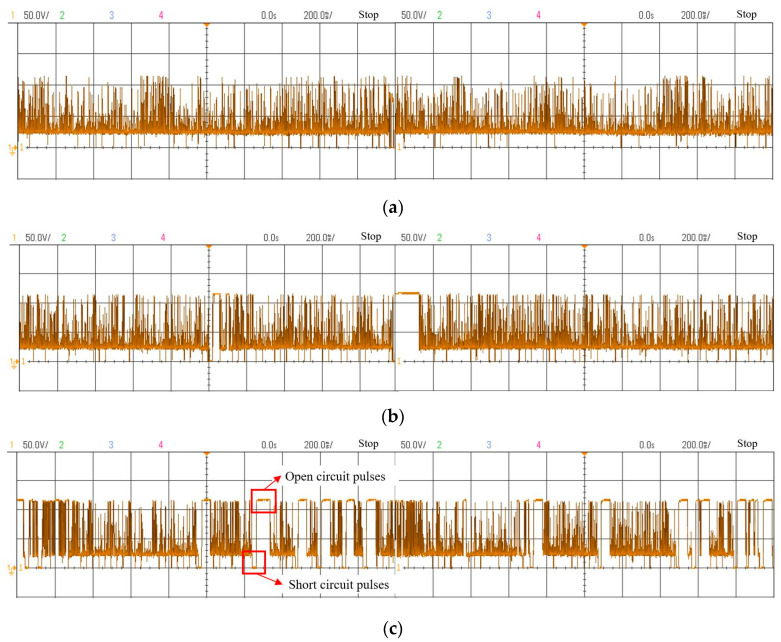
Effect of flushing pressure on voltage waveforms. (**a**) No flushing pressure; (**b**) Flushing pressure of 5 kPa; (**c**) Flushing pressure of 15 kPa.

From Figure 11a, when there is no flushing pressure, the erosion products cover the surface of the workpiece to be processed due to the poor discharge of the erosion products. Consequently, the proportion of the energy transferred to the workpiece by the discharge is reduced. Additionally, the accumulation of the erosion products in the discharge gap will increase the number of abnormal discharges, resulting in a large amount of carbon deposition in the workpiece cavity. When the flushing pressure increases to 5 kPa, the erosion products in the discharge gap are discharged in time. This enhances the stability of the inter-electrode discharge state, reduces the coverage of the erosion products, and improves the processing efficiency. When the flushing pressure is too high (10 kPa, 15 kPa), the movement of the erosion products in the discharge gap is too intense, which affects the discharge stability and is not conducive to the MRR. Figure 12 shows the gap voltage waveform collected by the oscilloscope (Agilent DSO-X2024A, Santa Clara, CA, USA) when the flushing pressure is 0, 5 kPa and 15 kPa. There is little difference in the number of effective pulses when the flushing pressure is 0 and 5 kPa. However, when the flushing pressure is increased to 15 kPa, it is obvious from the waveform that discharge points are sparse and dispersed, and the short circuit (the voltage between the two electrodes is almost 0) and open circuit (the voltage between the two electrodes is a no-load voltage) rates increase significantly. There is no discharge channel between the two poles during short and open circuits, and no energy is transmitted to the discharge gap, which is detrimental to processing. The decrease in effective pulse number leads to a decrease in processing efficiency. Therefore, with the increase in flushing pressure, the MRR increases first and then decreases.

The increased flushing pressure enhances the scouring effect of working liquid on the electrode surface, which is not conducive to forming a carbon black film. So, the EWR and REWR will increase. As shown in Figure 11e–h, the carbon black film on the bottom of the electrode becomes incomplete with increased flushing pressure. When the flushing pressure is 10 kPa, part of the copper matrix has been exposed on the bottom of the electrode. As seen from Figure 11a–d, when flushing is not applied, a carbon deposition layer is formed on the bottom surface of the workpiece cavity due to arc discharge, short circuit, etc. With the increase in flushing pressure, the carbon deposition layer disappears. But on account of the increase in electrode wear, residual material columns begin to be generated on the bottom surface of the workpiece cavity. Therefore, when using the slotted electrode for continuous internal flushing processing, the appropriate flushing pressure should be selected to ensure processing efficiency and reduce electrode wear.

## 4. Conclusions

In this study, the internal flushing slotted electrode was designed and prepared. The effects of pulse width, pulse interval, and flushing pressure on the MRR, the EWR, and the REWR were analyzed through single-factor experiments. Specific conclusions were drawn as follows:

1. With the increase in pulse width, the MRR increases first and then decreases, while the EWR and the REWR decrease gradually.
2. With the increase in pulse interval, the MRR decreases while the EWR and the REWR increase gradually.
3. With the increase in flushing pressure, the MRR increases first and then decreases, while the EWR and REWR increase gradually.
4. When the slotted electrode is used for continuous internal flushing EDM, the appropriate pulse width, flushing pressure, and smaller pulse interval can improve the MRR and reduce the EWR and the REWR.

## Figures and Tables

**Table 1:** Chemical composition list of high-speed steel (W9Mo3Cr4V).

Chemical Element	W	Cr	Mo	V	C	Si	S	P
Mass Fraction (%)	8.5–9.5	3.6–4.2	2.7–3.3	1.3–1.6	0.77–0.85	0.2–0.4	≤0.03	≤0.03

**Table 2:** Factor level table (peak current: 25.6 A; open circuit voltage: 100 V; reference voltage: 52 V).

Processing Parameters	Basic Level	Each Parameter Level
Pulse width (μs)	560	75, 320, 560, 750
Pulse interval (μs)	24	13, 24, 56, 100
Flushing pressure (kPa)	5	0, 5, 10, 15

## Data Availability

Not applicable.
